# Detection and Genomic Characterization of Novel Mammarenavirus in European Hedgehogs, Italy

**DOI:** 10.3201/eid3101.241084

**Published:** 2025-01

**Authors:** Barbara Di Martino, Federica Di Profio, Maria Teresa Capucchio, Ilaria Prandi, Serena Robetto, Giuseppe Quaranta, Giuseppina La Rosa, Elisabetta Suffredini, Fulvio Marsilio, Vito Martella, Vittorio Sarchese

**Affiliations:** University of Teramo, Teramo, Italy (B. Di Martino, F. Di Profio, F. Marsilio, V. Sarchese); University of Turin, Grugliasco, Italy (M.T. Capucchio, I. Prandi, G. Quaranta); S.S. Patologie della Fauna Selvatica, Istituto Zooprofilattico Sperimentale Piemonte, Liguria e Valle d'Aosta, Italy (S. Robetto); Istituto Superiore di Sanità, Rome, Italy (G. La Rosa, E. Suffredini); University of Bari Aldo Moro, Bari, Italy (V. Martella); University of Veterinary Medicine, Budapest, Hungary (V. Martella)

**Keywords:** viruses, *Arenaviridae*, mammarenaviruses, European hedgehog, Italy, zoonoses

## Abstract

Mammarenaviruses are noteworthy zoonotic pathogens, and the main reservoirs are rodent species. We report the detection of a novel mammarenavirus in 6/183 (3.3%) in necropsied European hedgehogs (*Erinaceus europaeus*) collected in Italy. The whole-genome sequence obtained for 4 strains revealed a marked genetic diversity but a monophyletic origin.

Mammarenaviruses are notable zoonotic pathogens. Several mammarenaviruses, including Lassa virus, Lujo virus, Junin virus, Machupo virus, Guanarito virus, and Chapare virus, are causative agents of severe viral hemorrhagic fevers (*1*). Mammarenaviruses are enveloped single-stranded RNA viruses classified in the genus *Mammarenavirus* within the family *Arenaviridae*, along with genera *Antennavirus*, *Hartmanivirus*, *Innmovirus*, and *Reptarenavirus* (*2*). The viral genome consists of 2 single-stranded ambisense RNA molecules, a small (S) segment (≈3,500 nt) that encodes the envelope glycoprotein precursor and the nucleoprotein (NP), and a large (L) segment (≈7,200 nt) encoding the zinc binding matrix protein (Z) and the viral RNA-dependent RNA polymerase (*2*). 

On the basis of their genetic, antigenic, and geographic relatedness, mammarenaviruses are divided into 2 groups: the New World (NW) group, which includes viruses indigenous to the Americas, and Old World (OW) group, which includes viruses indigenous to Africa, such as Lassa fever virus and the ubiquitous lymphocytic choriomeningitis virus (*3*). Except for Tacaribe virus, discovered in 2 Artibeus bat species (*4*), the natural hosts of arenaviruses are rodent species of the family *Muridae*; members of the subfamily *Murinae* are reservoirs of OW viruses, and rodents of the subfamilies *Sigmodontinae* and *Neotominae* are natural hosts of NW viruses (*2*). 

The diversity of arenaviruses is widely recognized to be the result of long-term coevolution with their natural hosts (*3*). However, with the increasing availability of molecular data from NW and OW viruses and their rodent reservoirs, the coevolutionary divergence hypothesis has been flanked by the evidence of arenavirus evolution through host switching (*5*). Those findings, alongside the discovery of mammarenaviruses in additional mammals, such as shrews (*Suncus murinus*) (*6*), plateau pikas (*Ochotona curzoniae*), (*7*), and, more recently, Northern white-breasted hedgehogs (*Erinaceus roumanicus*) (*8*), indicate other potential mammarenavirus reservoirs. In this study, we describe the detection and genetic characterization of a novel mammarenavirus in European hedgehogs (*E. europaeus*) in Italy.

## The Study

The study was performed on paired duodenal and liver samples collected in the Piedmont Region (Northwestern Italy) from 183 hedgehogs subjected to necropsy during 2018–2022. Of those, 146 animals were admitted to the Centro Animali Non Convenzionali of Turin University (Turin, Italy), whereas 37 additional animals were hospitalized at La Ninna, a rehabilitation center (Cuneo Prefecture, Italy). Samples were collected by authorized veterinarians following routine procedures from dead animals before the design of the study, in compliance with the Ethical Principles in Animal Research. Thus, ethics approval by an Institutional Animal Care and Use Committee was not deemed necessary.

**Figure 1:**
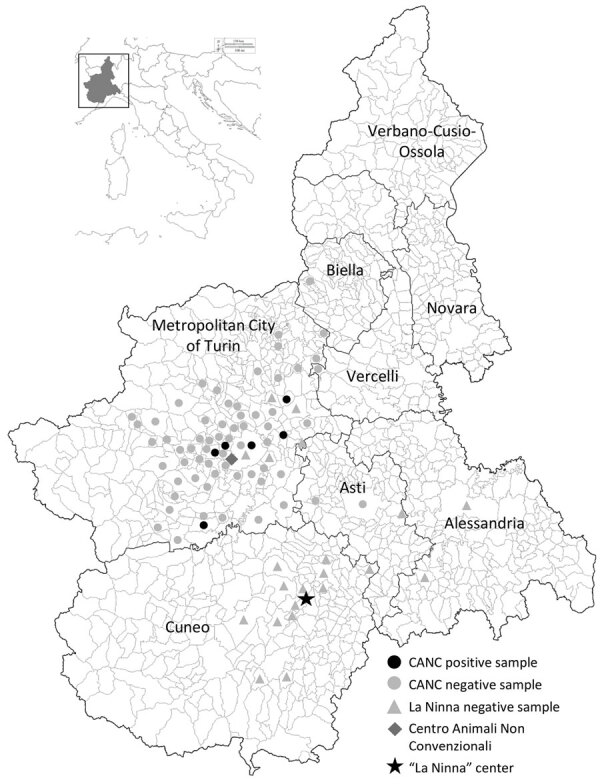
Municipalities of the Piedmont Region where hedgehogs were sampled in study of detection and genomic characterization of novel mammarenavirus in European hedgehogs, Italy. Inset shows location of Piedmont Region within Italy. CANC, Centro Animali Non Convenzionali.

During necropsy, we froze liver, duodenum, brain, spleen, kidney, and lung samples and transported them to the Department of Veterinary Medicine of Teramo (Teramo, Italy). To perform virological investigations, we homogenized liver and intestinal samples (10% wt/vol) in Dulbecco modified Eagle medium and extracted total RNA from the supernatant of the homogenates using TRIzol LS (Thermo Fisher Scientific, https://www.thermofisher.com). We conducted molecular screening by using genus-specific primers designed to amplify a conserved ≈390 nt region of the L gene of Lassa virus and related OW arenaviruses (*9*). We detected viral RNA in intestinal and liver specimens of 6/146 (4.1%) animals (identifications nos. 622/19, 1175/19, 1277/19, 328/22, 403/22, and 676/22), all rescued in Turin Prefecture from Centro Animali Non Convenzionali, whereas results from all La Ninna samples were negative (0/37) (Figure 1). Sanger sequencing of the amplicons generated in diagnostic reverse transcription PCR showed the highest identities (76.5%–77.8% nt) to the Alxa arenavirus RtDs-AreV-IM2014 (GenBank accession no. KY43289), prototype of the species *Mammarenavirus alashanense*, detected in 2018 in rectal swab samples collected from 3-toed jerboas (*Dipus sagitta*), a rodent species living in the deserts of the Inner Mongolia Autonomous Region of China (*10*), and to Mecsek Mountains arenavirus MEMV/MR1/2025/HUN (OP191655), which was identified in 2023 in fecal samples from Northern white-breasted hedgehogs in Hungary (*8*).

**Figure 2:**
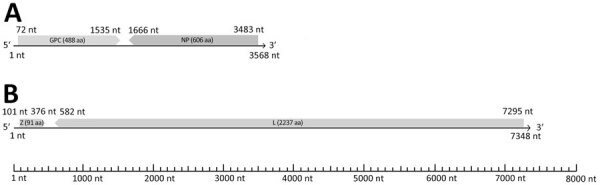
Schematic representation of the bisegmented genome organization of the *Erinaceus Europaeus* arenaviruses (EEAVs) detected in study of detection and genomic characterization of novel mammarenavirus in European hedgehogs, Italy, from 5′ to 3′ ends. A) The EEAV S genome segment (3,568 nt) coding for the putative GPC (1,464 nt) and NP (1,818 nt) proteins. B) The EEAV L genome segment (7,348 nt) encoding the putative Z (276 nt) and L proteins (6,714 nt). The proteins are shown in different shades of gray. Arrows indicate the direction of open reading frames. GPC, glycoprotein precursor; L, large; NP, nucleoprotein; S, small; Z, zinc-encoded matrix.

We subjected all positive samples to a sequence-independent enrichment protocol and sequenced them using the MinION Mk1C platform. We prepared libraries using the PCR Barcoding Expansion Kit 1–12 and the Ligation Sequencing Kit (all Oxford Nanopore Technologies, https://www.nanoporetech.com). Using the metaviromic pipeline of Genome Detective (*11*), we generated arenavirus-related contigs covering ≈80% of the complete L segment and ≈60% of the complete S segment from 4 liver samples. We used a primer walking strategy with specific primers designed to close the gaps between noncontiguous sequences to reconstruct the complete sequences of 4 *Erinaceus Europaeus* arenavirus (EEAV) strains designated EEAV/676/22/IT (GenBank accession nos. PP934155 for L segment, PP934161 for S segment), EEAV/1277/19/IT (PP934156 for L segment, PP934162 for S segment), EEAV/403/22/IT (PP934157 for L segment, PP934159 for S segment), and EEAV/1175/19/IT (PP934158 for L segment, PP934160 for S segment). The genome of the 4 strains showed the typical bisegmented structure in ambisense orientation (Figure 2). The L segment was 7,348 nt in length and contained 2 open reading frames of 276 nt and 6,714 nt, encoding the putative Z (91 aa) and L (2,237 aa) proteins, separated by a 205 nt noncoding region. As for other mammarenaviruses, in the Z protein, the N terminal myristoylation site for attachment of myristic acid **(G**_2_N**K**PT**K**VPSMQRT_14_), the centrally located RING domain (Y_50_LCL), and the 2 late domains **P**_83_[T/S]**AP** and **P**_87_P**Y,** were conserved (*12*). Also, the N terminal domain of the L protein contained the conserved active site motif characteristic of type II endonucleases (E_51_, D_89_, E_102_, K_115_, D_119_, and K_122_) (*13*). The S segment was 3,568 nt long and contained 2 open reading frames of 1464 nt and 1818 nt, coding for the putative glycoprotein precursor (488 aa) and NP (606 aa) proteins, with an intergenic region of 130 nt. On sequence analyses, the 4 EEAV strains shared 81.8%–93.9% nt identity in the S segment and 87.8%–93.5% nt identity in the L segment, indicating that they were variants of the same viral species. We compared deduced amino acid and nucleotide sequences with those of other representative mammarenaviruses. On pairwise sequence comparison, the 4 EEAVs were more closely related to the white-breasted hedgehog strain MEMV/MR1/2025/HUN (*8*), showing 73.2%–77.8% identity in the S segment (OP191656), 71.5%–72.0% in the L segment (OP191655), and 77.1%–78.9% in the NP amino acid sequence.

**Figure 3:**
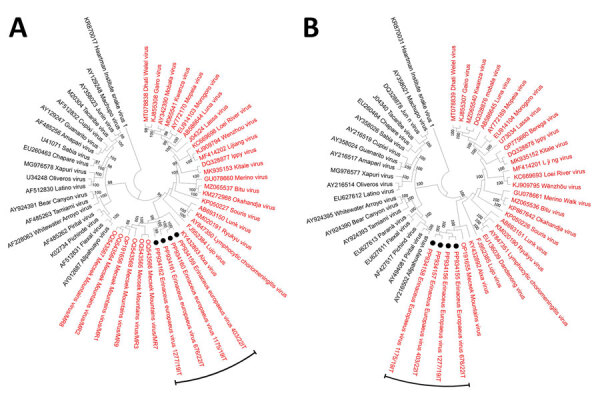
Neighbor-joining phylogenetic analyses based on nucleotide sequences of the complete small (A) and large (B) segments of mammarenavirus strains identified in study of detection and genomic characterization of novel mammarenavirus in European hedgehogs, Italy, and prototype species currently classified within the genus *Mammarenavirus*. The evolutionary distances were computed using the maximum composite likelihood method and are in the units of the number of base substitutions per site. A total of 1,000 bootstrap replicates was used to estimate the robustness of the individual nodes on the phylogenetic tree. Black circles indicate the four *Erinaceus europaeus* hedgehog arenavirus strains (EEAV/676/22/IT, EEAV/1277/19/IT, EEAV/403/22/IT, and EEAV/1175/19/IT) detected in this study. In both trees, the Haartman Institute snake virus 1, representative of the genus *Hartmanivirus*, is used as an outgroup. GenBank accession numbers are provided.

The cutoff values established by the International Committee on Taxonomy of Viruses (*2*) for arenavirus classification at the species level are >80% nt identity in the S segment and >76% nt identity in the L segment, with <12% aa difference in the NP protein. Accordingly, the hedgehog arenaviruses of this study meet the species demarcation criteria for classification as a novel mammarenavirus species. On phylogenetic analyses based on the S and L segments (Figure 3), the EEAV strains formed an independent clade within the OW mammarenaviruses, apart from the Mecsek Mountains viruses (*8*).

By assessing additional organs of the 6 positive animals, we detected EEAV RNA in brain (100%, 6/6), spleen (100%, 6/6), kidney (100%, 6/6) and lung (66.6%, 4/6) samples, suggesting systemic infection. Formalin-fixed paraffin-embedded tissue sections of the same organs of the 183 necropsied animals were also examined histologically. Overall, we observed no significant association between the histopathologic observed alterations and the presence of viral RNA, a feature consistent with the ability of arenaviruses to establish chronic infections with continuous virus production and little or no disease in their natural host (*14*).

## Conclusions

This study extends the knowledge of genetic diversity, host range, and geographic distribution of mammarenaviruses. Further investigations to establish whether hedgehogs represent underrecognized arenavirus reservoirs will be pivotal. European hedgehog is a synanthropic animal that can play a role in the ecology of potentially zoonotic viruses (*15*). Improved surveillance of at-risk persons, such as rescuers of ill or injured animals and operators of rescue centers, will be useful in investigating possible zoonotic exposure.
